# Facile: a command-line network compiler for systems biology

**DOI:** 10.1186/1752-0509-1-36

**Published:** 2007-08-03

**Authors:** Fernando Siso-Nadal, Julien F Ollivier, Peter S Swain

**Affiliations:** 1 Gene Network Sciences, 53 Brown Road, Ithaca, New York 14850, USA; 2 Centre for Non-linear Dynamics, Department of Physiology, McGill University, 3655 Promenade Sir William Osler, Montreal, Quebec H3G 1Y6, Canada

## Abstract

**Background:**

A goal of systems biology is the quantitative modelling of biochemical networks. Yet for many biochemical systems, parameter values and even the existence of interactions between some chemical species are unknown. It is therefore important to be able to easily investigate the effects of adding or removing reactions and to easily perform a bifurcation analysis, which shows the qualitative dynamics of a model for a range of parameter values.

**Results:**

We present Facile, a Perl command-line tool for analysing the dynamics of a systems biology model. Facile implements the law of mass action to automatically compile a biochemical network (written as, for example, E + S <-> C) into scripts for analytical analysis (Mathematica and Maple), for simulation (XPP and Matlab), and for bifurcation analysis (AUTO). Facile automatically identifies mass conservations and generates the reduced form of a model with the minimum number of independent variables. This form is essential for bifurcation analysis, and Facile produces a C version of the reduced model for AUTO.

**Conclusion:**

Facile is a simple, yet powerful, tool that greatly accelerates analysis of the dynamics of a biochemical network. By acting at the command-line and because of its intuitive, text-based input, Facile is quick to learn and can be incorporated into larger programs or into automated tasks.

## Background

Biology is becoming a quantitative science. Driven by the introduction of the Systems Biology Markup Language [1], an XML-based language for communicating models between different tools, a large number of software packages now exist that simulate networks of interacting genes and proteins [2]. The majority of these tools aim to provide a modelling platform for experimental biologists. Often a model can be input via a GUI-based interface, which links to a numerical integration routine for generating time series of protein concentrations [3-7].

The quantification of the life sciences is also attracting to systems biology members of the mathematical biology and biophysics communities. These scientists are usually mathematically and computationally sophisticated and already have standard computational tools for investigating many questions posed by systems biology. They typically work at the command-line in a UNIX programming environment and prefer text-based rather than GUI-based methods of model entry.

Here we introduce Facile, a network compiler for investigating the dynamics of biochemical networks. Facile is aimed specifically at mathematicians, engineers, and physicists working in systems biology. From a textual description, Facile compiles the biochemical network into formats for the standard computational tools used in studies of non-linear dynamics. Facile has several advantages: (1) with one command invocation, it links to all the standard analysis tools used by the mathematical biology and non-linear dynamics communities; (2) it has an intuitive, text-based input, with a format for representing chemical reactions similar to that adopted by most textbooks, making it easy to learn; (3) it is command-line oriented and therefore, like any UNIX command, can be easily incorporated into automated tasks or other software; and (4) it outputs SBML and therefore allows, for example, a mathematical biologist familiar with UNIX to use the SBML tool set with minimal effort.

Biochemical networks are non-linear systems. Within non-linear dynamics, three methods have been developed to analyse non-linear behaviour: analytical approaches, simulation, and bifurcation analysis.

Facile greatly aids bifurcation analysis. It compiles a version of the model from text into the C programming language to interface directly with AUTO, a standard bifurcation analysis tool [8]. It automatically  generates reaction rates using the law of mass action and  automatically finds any mass conservations present in the system. Such mass conservations need to be explicitly identified for bifurcation analysis software. Otherwise, different initial conditions change the total concentration of a conserved chemical species in the system and lead to lines of fixed points where only a single fixed point is expected. Other packages that interact with AUTO typically require the user to initially specify the model in this reduced format. For networks of the size often modelled in systems biology, such a specification is laborious and would be necessary every time the model is modified.

In addition, Facile converts a network model into a format for analytical analysis by computer algebra packages – Mathematica (Wolfram Research, Champaign, IL) or Maple (MapleSoft, Waterloo, ON) – and into a format for simulation – XPP [9] or Matlab (The Mathworks, Natick, MA). Through its XPP output, Facile interfaces with XPPAUT, a GUI-based, although not as flexible, version of AUTO with XPP.

Facile is not intended to compete with SBML, but is complementary through its command-line character and its ability to convert an intuitive, text-based form of a model into SBML. Unlike other SBML converters, such as CellDesigner [10], JDesigner [11], and SBMLeditor [12], Facile is not GUI-based and therefore can be incorporated into larger, automated tasks via, for example, UNIX piping and scripting. Although Facile is the only command-line compiler that produces a reduced version of a systems biology model in C for AUTO of which we are aware, its other capabilities can be performed by software already developed. Nevertheless the user has to install a significant amount of code, learn how to use the code, and needs to know from the hundreds of SBML compatible packages the appropriate ones to install. A different package would be required for each task, whereas with Facile they are carried out in a single invocation, with the appropriate command-line options. For example, MathSBML could be used to link to Mathematica [13], the SBMLToolbox to link to Matlab [14], JigCell to link to XPP [15], and Oscill8 could be used for bifurcation tracking [16]. More software would be needed to convert the initial text-based version of the model into rate equations and then into SBML, such as Dizzy [6], and to find any mass conservations, such as COPASI [7].

## Implementation and results

Facile is modular and coded in Perl using an object-oriented methodology with a distinct module to generate each output. A new output option or a modification of an existing option can be easily implemented.

Facile uses the method proposed by Sauro and Ingalls [17] to automatically find mass conservations in any model. Mass conservations reduce the number of independent variables in the system and must be explicitly identified for bifurcation analysis.

### Input file

Facile's input is simple: a system of chemical reactions. Facile automatically calculates reaction rates using the law of mass action. Therefore, for example, the Michaelis-Menten reaction is specified as

E + S <-> C ; f = 1.6e7; b = 6

C -> P + E ; k = 0.15

The association rate between the enzyme, E, and the substrate, S, is 1.6 × 10^7 ^M^-1 ^s^-1^, and the complex, C, dissociates into the product, P, and enzyme with rate 0.15 s^-1^. Backward reactions can also be written explicitly, e.g.

E + S <- C ; b = 6

Sources, for example gene expression, and sinks, for example protein degradation, are denoted by null:

null -> A ; s = 10

A -> null ; d = 0.1

implying that protein A is produced at a rate of 10 s^-1 ^and degrades at a rate of 0.1 s^-1^.

Facile also allows explicit specification of reaction velocities, time-varying system parameters, and effective rate expressions, such as Hill functions. A thick arrow, =>, indicates a reaction velocity and overrides the law of mass action. The reaction velocity may be a constant or an expression enclosed in double quotes. For example, the Michaelis-Menten equations could be specified as

E + S < = > C; f = "1.6e7*E*S"; b = "6*C"

C = > P + E; k = "0.15*C"

or as

S = > P; f = "S*V/(K + S)"

in the quasi-steady state approximation. Variables can also be defined, such as

variable V = 0.15*0.2

variable K = (0.15+6)/16

for the quasi-steady state approximation with a total enzyme concentration of 0.2 *μ*M. Once a variable has been specified, its value is available throughout the rest of the input file.

Time can be included in a reaction rate by using t. For example, the expression of a gene may be periodically modulated by the cell cycle:

null -> A ; s = "10*(1+cos(2*pi*t/2400))"

for a cell cycle of 40 minutes or 2400 seconds.

To run simulations, initial concentrations are also needed. Concentrations are specified beneath the INIT keyword in the input file. Any concentration not specified is set to zero. For the Michaelis-Menten example, we have

INIT

E = 0.2 uM

S = 3

implying that P is initially zero, S is initially 3 M, and E is initially 0.2 *μ*M. The substrate could also be initialized as S = 3 M.

### Output for analytical analysis

Mathematica and Maple are two standard computer algebra packages. They can be used to derive analytical expressions for steady-state concentrations, for the eigenvalues determining the stability of the steady-state, or to evaluate perturbation expansions, such as the method of multiple scales.

The command

facile.pl -M model

converts the textual input for the Michaelis-Menten system shown earlier (and contained in the file model) into Mathematica format. Since Facile automatically applies the law of mass action, it generates the expressions

dEdt = + b C - f E S + k C ;

dSdt = + b C - f E S ;

dCdt = + f E S - b C - k C ;

dPdt = + k C ;

These expressions can be cut-and-pasted or loaded into Mathematica and then algebraically manipulated. For example, they can be solved for the quasi-steady state solution. The -L option generates an equivalent format for Maple.

### Output for simulation

Analytical solutions are usually only possible for an approximate form of a model, if at all. Facile provides an ordinary differential equation version of the model for input to Matlab and Octave [18], via the -m option, or XPP, via the -x option. These software packages provide tools for numerically integrating ordinary differential equations and visualizing their solutions. Two files are created for Matlab: one describing the model as a system of ordinary differential equations and another (driver) file for the user. An ode file is created for XPP.

Nevertheless, intracellular molecular numbers are often small, and significant stochastic fluctuations can exist in biochemical networks [19]. Through its SBML output, Facile can interface with many stochastic simulators, but it is also supplied with EasyStoch, a stochastic simulator written in C that supports time-varying kinetic rates. EasyStoch is based on the Gibson-Bruck version [20] of the Gillespie algorithm [21].

### Output for bifurcation analysis

A bifurcation analysis is frequently used to show how the qualitative behaviour of a system changes when parameter values are altered. It is particularly important in systems biology where many parameters are unknown and where the dynamical behaviour of the systems is often expected to be robust to parameter changes [22].

AUTO is a standard tool for numerical bifurcation analysis [8]. Like any bifurcation analysis tool, AUTO requires all mass conservations in the system to be explicitly written and the model to be presented in a reduced form.

Facile automatically finds any mass conservations and incorporates them into a reduced system model which is only a function of the independent variables (those chemical species that are not related by mass conservations). For example, consider again the Michaelis-Menten system. It has two mass conservations: the total amount of enzyme is conserved, in either the E or C form, and the total amount of substrate is conserved, in either the S, C, or P form. Running Facile with the -rM option, produces a Mathematica version of the model in the reduced form:

E = - C + E_tot ;

S = - P - C + S_tot ;

dCdt = + f E S - b C - k C ;

dPdt = + k C ;

where E_tot is the total amount of enzyme and S_tot is the total amount of substrate. Facile determines the numerical value of these two constants from the initial conditions specified in the input file.

Once the mass conservations have been identified, there is not a unique reduced form for the system. For example, C and P could be related to E_tot and S_tot in the Michaelis-Menten example, and E and S would then be the independent variables. In the input file to Facile, the user can specify the chemical species that should be independent and those that should be dependent. Any chemical species not specified will have its dependency assigned automatically. The MOIETY section in the input file is used for these specifications. For example,

MOIETY

dependent E, S

ensures that the Michaelis-Menten equations are reduced to the form above. So, too, does

MOIETY

independent C, P

A combination of independent and dependent variables is also valid.

Although XPPAUT does allow bifurcation analysis (and is supported by the -x option in Facile), the complex biochemical networks studied in systems biology usually require the stand-alone version of AUTO because it allows the user to tune more of AUTO's parameters. Via its -a option, Facile produces a C version of the reduced model in the format expected by AUTO. AUTO also needs to know the parameters to be varied for the bifurcation analysis. These are specified in the BIFURC_PARAM section of the Facile input file and are incorporated into the C version of the reduced model. For example, if the user wished to investigate whether the system bifurcates as the association rate, f, and dissociation rate, b, for the Michaelis-Menten reaction are varied, Facile requires

BIFURC_PARAM

f, b

in its input file.

As a starting point for generating the bifurcation diagram, bifurcation analysis software uses the concentrations of the chemical species in a system at an attractor of the system. Usually a starting point is found by integrating the system to steady-state. Facile with its -A option will automatically load the values of the steady-state concentrations from a text file, generated by Matlab or XPP, and incorporate them into the C version of the model required by AUTO.

Facile greatly speeds up bifurcation analysis of biochemical models. Here we illustrate the steps that a user could follow to set up a bifurcation analysis. Facile can be used to generate both the simulation file and the C AUTO file. The simulation file is useful for finding the initial point for the bifurcation analysis and for exploring the dynamics of the model in different regions of the bifurcation diagram. As an example, we will use XPP as the integrator. We specify the model as a system of chemical reactions in the text file model. For XPP output, we run facile.pl -rx model at the command-line to generate model.ode. This XPP file is a reduced form of the model in the form of ordinary differential equations: rates are automatically calculated by the law of mass action and any mass conservations are found and used to reduce the model to the minimum number of independent variables. We then integrate the model to a steady-state and use the XPP data browser to save the corresponding time course data to a space-delimited text file, output.dat. Alternatively, we could run XPP without an interface with the command xppaut model.ode -silent. This command runs XPP and saves the time series generated in output.dat. By running facile.pl -aA output.dat model, we create a C version of the model, which includes the steady-state concentrations taken from the last time point of output.dat. We then directly load into AUTO the model.c file created. Matlab could also be used to integrate the model equations (with the -rm option in Facile). In summary, the single-line command facile.pl -rx model; xppaut model.ode -silent; facile.pl -aA output.dat model builds both the C model expected by AUTO and an XPP simulation file.

### SBML export

SBML export is enabled by the -S option and uses the libSBML package, which must also be installed.

### Miscellaneous

As mentioned, variables or parameters can be defined in the Facile input file using the variable command. The input file also has a CONFIG section where if desired the user can specify the time interval to run simulations, the particular differential equation solver to be used by Matlab, and XPP configuration commands. See the online Facile manual.

## Discussion and conclusion

Facile is a tool to quickly create models of biochemical networks and to analyse their dynamics. Although specifically aimed at systems biologists from the non-linear dynamics community, Facile should be useful to any computational biologist modelling biochemical networks. It links directly to the standard analysis tools used for non-linear systems and indirectly to many more via SBML.

A major feature of Facile is its ability to generate a reduced form of a model in C for the bifurcation analysis software AUTO. Bifurcation analysis gives the global behaviour of non-linear systems for ranges of parameter values. It is particularly important in systems biology where many parameter values are unknown or are only known approximately. It is essential for bifurcation analysis that any mass conservations in a model are identified and used to write the model in a form with a minimum number of independent variables. In the Michaelis-Menten system, for example, the sum of the free enzyme and complex is equal to the total amount of enzyme. If this mass conservation is not identified, varying the initial conditions for the free enzyme and for the complex, will create a system with a different amount of total enzyme that has its own steady-state. AUTO will therefore find a line of steady-states rather than the expected single steady-state. Facile automatically identifies and uses mass conservations to produce a reduced form of any model.

The input to Facile is a text file of chemical reactions written in a notation very similar to that used by most undergraduate text books. Facile automatically calculates reaction rates and generates rate equations. Exploring changes in model structure with Facile is therefore straightforward. Chemical reactions can be added to or removed from the input file, and Facile ensures that the rate equations are generated consistently.

By using a text file as input and because it operates at the command-line, Facile can be run automatically and embedded into larger programs. In addition, there are several problems in systems biology where a GUI interface would not be suitable to build a model. In signal transduction, for example, the number of proteins that can both exist in different phosphorylated states and bind together in complexes rapidly leads to a large number of distinct chemical species. An example is the Ste5 scaffold protein in the yeast pheromone response pathway which is involved in 1300 different chemical species [23]. A GUI based approach to compile this network is infeasible.

Written in Perl, Facile fits naturally into the UNIX programming environment. It is an easily learned addition to a systems biologist's toolbox.

## Availability and requirements

Facile runs in any programming environment that can support Perl, such as Linux, Mac OS X, and Windows. Facile and its manual are available and can be used online at  [see also Additional file 1]. We use the libSBML library for the SBML export function [1] and standard modules from the Comprehensive Perl Archive Network [24].

## Authors' contributions

FSN and JFO wrote Facile; PSS wrote an early version. JFO wrote the online manual. FSN, JFO, and PSS wrote the paper.

## Supplementary Material

Additional file 1 Facile source code. the Perl source code for the Facile command-line tool. Click here for file

## References

[B1] The Systems Biology Markup Language. http://sbml.org.

[B2] Alves R, Antunes F, Salvador A (2006). Tools for kinetic modeling of biochemical networks. Nat Biotechnol.

[B3] Loew LM (2002). The Virtual Cell project. Novartis Found Symp.

[B4] Shapiro BE, Levchenko A, Meyerowitz EM, Wold BJ, Mjolsness ED (2003). Cellerator: extending a computer algebra system to include biochemical arrows for signal transduction simulations. Bioinformatics.

[B5] Dhar P, Meng TC, Somani S, Ye L, Sairam A, Chitre M, Hao Z, Sakharkar K (2004). Cellware-a multi-algorithmic software for computational systems biology. Bioinformatics.

[B6] Ramsey S, Orrell D, Bolouri H (2005). Dizzy: stochastic simulation of large-scale genetic regulatory networks. J Bioinform Comput Biol.

[B7] Hoops S, Sahle S, Gauges R, Lee C, Pahle J, Simus N, Singhal M, Xu L, Mendes P, Kummer U (2006). COPASI-a COmplex PAthway SImulator. Bioinformatics.

[B8] AUTO: Software for continuation and bifurcation problems in ordinary differential equations. http://indy.cs.concordia.ca/auto.

[B9] XPPAUT. http://www.math.pitt.edu/~bard/xpp/xpp.html.

[B10] Cell Designer. http://www.systems-biology.org/cd.

[B11] JDesigner. http://jdesigner.org.

[B12] Rodriguez N, Donizelli M, Le Novere N (2007). SBMLeditor: effective creation of models in the Systems Biology Markup language. BMC Bioinformatics.

[B13] Shapiro BE, Hucka M, Finney A, Doyle J (2004). MathSBML: a package for manipulating SBML-based biological models. Bioinformatics.

[B14] Keating SM, Bornstein BJ, Finney A, Hucka M (2006). SBMLToolbox: an SBML toolbox for MATLAB users. Bioinformatics.

[B15] Vass M, Allen N, Shaffer CA, Ramakrishnan N, Watson LT, Tyson JJ (2004). The JigCell model builder and run manager. Bioinformatics.

[B16] Oscill8 Dynamical Systems Toolset. http://oscill8.sourceforge.net.

[B17] Sauro HM, Ingalls B (2004). Conservation analysis in biochemical networks: computational issues for software writers. Biophys Chem.

[B18] Octave. http://www.octave.org.

[B19] Kaern M, Elston TC, Blake WJ, Collins JJ (2005). Stochasticity in gene expression: from theories to phenotypes. Nat Rev Genet.

[B20] Gibson MA, Bruck J (2000). Efficient exact stochastic simulation of chemical systems with many species and many channels. J Phys Chem A.

[B21] Gillespie D (1977). Exact stochastic simulation of coupled chemical-reactions. J Phys Chem.

[B22] Kitano H (2004). Biological robustness. Nat Rev Genet.

[B23] Lok L, Brent R (2005). Automatic generation of cellular reaction networks with Moleculizer 1.0. Nat Biotechnol.

[B24] Comprehensive Perl Archive Network. http://www.cpan.org.

